# Injury incidence, patterns, and severity in cameroon professional Men’s Football: A prospective injury surveillance study

**DOI:** 10.1371/journal.pone.0348717

**Published:** 2026-05-07

**Authors:** Gilbert Mua Akwa, Tankeng Leonard Tanko, Njowe Serge Ludwig, Ange Veronique Ngo Bilong, Nana Chunteng Theophile

**Affiliations:** 1 Department of Clinical Sciences, Faculty of Medicine and Biomedical sciences, University of Yaounde I, Center, Cameroon; 2 Department of Surgery, Faculty of Medicine and Pharmaceutical Sciences, University of Douala, Littoral, Cameroon; 3 Department of Research, SPORTSMED Foundation, Littoral, Cameroon; 4 Medical Commission, Cameroon Football Federation, Center, Cameroon; 5 Department of Surgery, Faculty of Health Sciences, University of Buea, Southwest, Cameroon; Universidade de Evora, PORTUGAL

## Abstract

**Background:**

Football injuries are a major source of morbidity among athletes, imposing health and economic burdens on teams and systems. While extensive surveillance exists in Europe, data from sub-Saharan Africa remain scarce. This study described the incidence, patterns, severity, and contextual factors of match-related injuries among professional male footballers in Cameroon.

**Methods:**

A prospective cohort design was conducted during the 2023/2024 Cameroon Elite 1 and Elite 2 league playoff tournaments. Match-day medical officers recorded all medical-attention injuries using standardized consensus-based forms [1]. Injury incidence was calculated per 1,000 player-hours, and descriptive analyses summarized injury characteristics. Ethical approval was obtained from the University of Douala and authorization from the Cameroon Football Federation.

**Results:**

Across 139 matches, 171 injuries were recorded giving an incidence of 37.3 per 1,000 player-hours. Elite 2 players had more than double the rate of Elite 1 players (53.6 vs. 26.3). Lower-limb injuries (71.9%) predominated, mainly affecting the thigh (33.9%) and lower leg (17.5%). The most common diagnoses were cramps (20.5%), contusions (19.9%), and sprains (17.5%). Severe injuries (>28 days) represented 20.5%, with tears, fractures, and concussions most frequent. Nearly half injuries occurred in the final 30 minutes of play, and midfielders and attackers sustained 68% of all injuries.

**Conclusion:**

This first surveillance study in Cameroon reveals a high match injury burden, particularly in lower-tier leagues. The late-match clustering supports fatigue-related mechanisms, emphasizing the need for targeted prevention focusing on conditioning, workload regulation, and medical readiness in African football.

## Introduction

Injury in football is defined as any physical complaint sustained by a player during a match or training session, irrespective of the need for medical attention or absence from future participation. Injuries that result in the player receiving medical attention are termed medical-attention injuries, whereas those causing absence from subsequent training or matches are considered time-loss injuries [1].

Professional footballers are at high risk of injury, which can negatively impact player health, team performance, and career longevity. Reliable injury surveillance is essential to guide prevention strategies by identifying incidence, mechanisms, and risk factors [2,3].

Extensive surveillance has been conducted in major international tournaments such as the FIFA World Cup [4–6]. In Europe, the UEFA Elite Club Injury Study has provided long-term monitoring across top clubs, demonstrating higher injury incidence during matches compared to training, and the predominance of lower-extremity injuries [7]. Similar studies in Sweden and Finland have offered prospective data on professional leagues, highlighting differences between acute and overuse injuries, as well as the influence of season length and match congestion [8,9].

In Africa, injury surveillance in football is less well established than in Europe and other high-resource settings. Data from the 2021 Africa Cup of Nations (AFCON) reported muscle strains and contact injuries as the most common injury types [10]. Similarly, studies in youth football from South Africa have documented high injury rates, particularly involving lower-limb strains and contact mechanisms [11]. In Cameroon, Ghana, and comparable settings, several studies have examined injury patterns and risk factors in youth and academy players; however, systematic injury surveillance data from professional domestic leagues remain largely unavailable [12–14]. Existing surveillance efforts in sub-Saharan Africa have predominantly focused on zonal or tournament settings, such as the COSAFA Women’s Championship, which provided important insights into injury burden and medication use in female football [ 15,16]. Nevertheless, surveillance data from professional men’s domestic leagues in the region are scarce.

Given that competition schedules, playing conditions, and access to medical resources differ substantially from those in European and other high-resource environments, injury surveillance data from African professional leagues are essential to inform injury prevention and management strategies. To address this gap, we conducted a prospective injury surveillance study to investigate the incidence, patterns, and immediate on-field management of injuries in Cameroon’s men’s professional football league.

## Methods

### Study design and setting

This prospective cohort study was conducted during the 2023/2024 Cameroon Elite 1 and Elite 2 league playoffs, held between March and May 2024. Injury surveillance was limited to official playoff matches. The playoffs were held across three regions of Cameroon:

Yaoundé (Elite 1 – Playoffs Down): Yaoundé Omnisport Annex and Olembe Annex Stadium

Douala (Elite 1 – Playoffs Up): Mbappé Leppe and Bonamoussadi Stadium.

West Region (Elite 2 – Playoffs Up & Down): Stade Bamendzi, Mbouda, and Bafang Stadium

### Study population and sampling

All male professional footballers who participated in the 2023/2024 Elite 1 and Elite 2 playoff matches were eligible for inclusion. Injury data were prospectively collected by corresponding match-day medical officers appointed by the Cameroon Football Federation. During each match, injuries that received medical attention were recorded on standardized forms, in line with the consensus statement on football injury surveillance [1]. Data was collected from the anonymised match day injury surveillance reports by match day officers from March 1^st^ – May 31^st^ 2024. No participant could be identified from his collected data as this was completely anonymous.

### Ethical considerations

The study received ethical clearance from the University of Douala (No. 4297 IEC-UD/06/2022/T) who waived consent since our methodology involved collecting data from recorded match day injury reports. Administrative authorization from the Cameroon Football Federation (No. 0701/FECAFOOT/SG/ASG/022) to access their anonymised match day injury surveillance report data. Confidentiality was maintained by assigning serial numbers to participants instead of using personal identifiers. Data was collected from the anonymised match day injury surveillance reports by match day officers. All data were coded and handled following recommended guidelines to ensure anonymity.

### Study procedure

Following ethical and administrative approvals, eligible player records were extracted from official match reports. Match-day medical officers prospectively documented all injuries using standardized anonymous consensus based forms during each game. Injury severity (time-loss) was estimated using a diagnosis-based approach. Match-day medical officers documented the suspected injury diagnosis at pitch side and, where applicable, incorporated findings from imaging performed on match day for players referred to designated hospitals. Diagnoses were subsequently mapped to anticipated time-loss categories using international consensus definitions and typical return-to-play timelines reported in the football injury literature. All forms were collected immediately after each game then coded and entered into a database to ensure confidentiality. Injury surveillance was restricted to official playoff matches, as no centralized or standardized system exists for capturing training exposure or training-related injuries across participating clubs, precluding reliable collection of training injury data.

### Statistical analysis and definition

Data were entered into Microsoft Excel 2016 and analysed using SPSS version 25.0. Categorical variables were summarized as frequencies and percentages, and continuous variables were presented as means with standard deviations.

## Results

### Injury incidence

A total of 139 matches from the 2023/24 Cameroon Elite 1 and Elite 2 playoff tournament were monitored, corresponding to 4,587 player-hours of exposure. These included 28 matches in Elite 1 Playoffs Up, 55 in Elite 1 Playoffs Down, and 56 in the Elite 2 Playoffs. Across these matches, 171 medical-attention injuries were recorded, yielding an overall incidence of 37.3 injuries per 1,000 player-hours.

When stratified by league, Elite 1 recorded 72 injuries over 2,739 player-hours (26.3 injuries per 1,000 player-hours), while Elite 2 recorded 99 injuries over 1,848 player-hours (53.6 per 1,000 player-hours) more than double the Elite 1 rate. This indicates a substantially higher injury risk in the lower-tier competition, possibly reflecting differences in match intensity, pitch quality, and medical support availability.

The mean age of injured players was 23.4 ± 2.9 years, with two-thirds of injuries occurring between 21 and 26 years of age typical peak playing years. Midfielders sustained the largest proportion of injuries (34.5%), closely followed by attackers (33.3%), while defenders accounted for 25.7%, and goalkeepers were least affected (6.4%). This pattern reflects the high physical demands of midfield and attacking roles, which involve repeated accelerations, directional changes, and frequent player contact.

### Time of injury

Injury occurrence varied across match periods, with the highest proportion recorded in the final stages of play. The 76–90 min period accounted for 21.6% of all injuries, followed by 19.9% between 61–75 min, together representing 41.5% of all injuries and indicating a clear late-match clustering. Fewer injuries occurred earlier in games (17.5% between 31–45 min, 15.2% between 0–15 min, and 12.9% each between 16–30 min and 46–60 min). This temporal pattern highlights an increased injury risk toward the end of matches, likely reflecting fatigue accumulation and reduced neuromuscular control.

### Injury type and anatomical locations

Injuries predominantly involved the lower extremities, particularly the thigh (33.9%), lower leg (17.5%), ankle (10.5%), and knee (7.6%). Upper-limb and trunk injuries were less frequent, while head and face injuries together accounted for 11.7% of cases. Overall, lower-limb injuries represented 71.9% of all cases, reaffirming the dominant burden of lower-extremity trauma in football. (Table 1).

**Table 1:** Injury type and anatomical locations.

Main Grouping	Category	n	%
**Head and neck**	Head/Face	20	11.7
	Neck/Cervical spine	2	1.2
**Upper limbs**	Shoulder/Clavicle	2	1.2
	Upper arm	1	0.6
	Elbow	2	1.2
	Forearm	4	2.3
	Wrist	1	0.6
	Hand/Finger/Thumb	10	5.8
**Trunk**	Sternum/Ribs/Upper back	2	1.2
	Lower back/Pelvis/Sacrum	4	2.3
**Lower limbs**	Hip	1	0.6
	Groin	2	1.2
	Thigh	58	33.9
	Knee	13	7.6
	Lower leg/Achilles tendon	30	17.5
	Ankle	18	10.5
	Foot/Toe	1	0.6
**Total**		**171**	**100**

The most frequent diagnoses were cramps (20.5%), contusions (19.9%), and sprains (17.5%), followed by lacerations (12.3%), muscle strains (11.7%), and tears (7.6%). Less frequent but clinically significant diagnoses included concussions (4.1%), fractures (2.9%), and dental injuries (2.3%) (Table 2).

**Table 2:** Injury categories and diagnoses by player position.

Injury category	Specific diagnosis	Attackers	Midfielders	Defenders	Goalkeepers	(n)	%
Muscle / Tendon	Cramps	14	13	8	0	35	20.5
	Strains	8	7	5	0	20	11.7
	Tears	2	6	3	2	13	7.6
**Subtotal – Muscle/Tendon**		**24**	**26**	**16**	**2**	**68**	**39.8**
Joint / Ligament	Sprains	12	11	5	2	30	17.5
	Dislocations	1	0	1	0	2	1.2
Subtotal – Joint/Ligament		13	11	6	2	32	18.7
Contusions	Contusions	8	15	9	2	34	19.9
Skin	Lacerations	6	4	9	2	21	12.3
Bone	Fractures	3	2	0	0	5	2.9
Nervous System	Concussions	2	1	2	2	7	4.1
Dental	Dental	1	0	2	1	4	2.3
**Total**		**57**	**59**	**44**	**11**	**171**	**100**

After classification, muscle and tendon injuries were the largest diagnostic group (39.8%), driven mainly by cramps, strains, and tears. Contusions accounted for 19.9%, followed by joint and ligament injuries (18.7%) and skin injuries such as lacerations (12.3%). Bone (2.9%), nervous system (4.1%), and dental (2.3%) injuries were relatively uncommon but often severe (Table 2). This distribution is consistent with patterns observed in professional football globally, where soft-tissue and overuse injuries dominate.

### Injury severity

Severity analysis showed that 136 injuries (79.5%) were classified as not severe and 35 (20.5%) as severe (time-loss > 28 days). The burden of severe injuries was unevenly distributed: only 23.3% of sprains and 5.0% of muscle strains were severe, whereas all structural diagnoses including tears, fractures, and concussions resulted in prolonged absence. Goalkeepers, though less frequently injured, sustained the highest proportion of severe cases (45.5%), reflecting their greater exposure to high-impact mechanisms. The proportion of severe injuries was comparable between leagues (Elite 1: 20.8%; Elite 2: 20.2%), suggesting that competition level did not markedly influence injury severity.

### Player position and injury

Cross-analysis of type, position, and timing revealed distinct risk profiles. Cramps disproportionately affected attackers late in matches (Table 2), consistent with fatigue-related mechanisms. Sprains clustered around half-time particularly in attackers and were associated with greater severity. Defenders were more prone to early-match lacerations, indicating a predominance of contact-related trauma during initial phases of play.

Overall, Cameroon’s men’s professional football playoffs demonstrated a high injury incidence compared with European elite leagues, with a disproportionate burden in the Elite 2 division, clustering of injuries in the final 30 minutes, and a concentration of severe cases among structural injuries (tears, concussions, fractures). These findings underscore the need for context-specific prevention strategies, focusing on fatigue management, contact protection, and improved medical preparedness within sub-Saharan African football.

## Discussion

This study presents the first prospective injury surveillance data from Cameroon’s professional men’s football leagues, revealing a notably high overall injury incidence (37.3 injuries per 1,000 player-hours) and a predominance of soft-tissue and lower-limb trauma. The findings indicate a substantial burden of match-related injuries, particularly within the lower-tier Elite 2 league. These results contribute valuable regional data to global football injury epidemiology and emphasize the need for context-specific prevention strategies in Cameroonian professional football and similar environments.

### Injury incidence and context

The overall injury incidence reported in this study is higher than that documented in European elite football. The UEFA Elite Club Injury Study, which monitored top-tier teams over multiple seasons, recorded match injury incidences ranging from 24 to 30 per 1,000 player-hours [7,17]. Similarly, the Asian Football Confederation (AFC) surveillance reported a match injury rate of approximately 19.2 per 1,000 player-hours [18], and another report from the first division league in Qatar revealed an incidence of 14.5 per 1000 hours [19], while South American professional leagues have shown comparable values of around 20.9 per 1,000 player-hours [20]. Also, a study done in the South African professional male league among reported a match injury incidence of 24.8 per 1000 hours [21]. The notably high overall injury incidence in Cameroonian professional football particularly within the Elite 2 division (53.6 per 1,000 player-hours) likely reflects contextual challenges such as suboptimal pitch conditions, limited availability of qualified medical personnel in these teams, and disparities in conditioning and tactical demands between divisions. Similar patterns have been described in lower-tier European and South American leagues, where infrastructural and medical disparities have been linked to elevated injury rates [1,21]. Despite methodological differences, these findings reinforce that professional footballers competing in resource-limited environments face a greater risk of preventable injury, emphasizing the importance of tailored prevention and medical capacity-building efforts.

### Anatomical distribution and injury type

The dominance of lower-limb injuries (71.9%) in this study is consistent with international findings, including those from UEFA and FIFA tournaments, where over two-thirds of all injuries affect the lower extremities [4,22]. The thigh (33.9%) and lower leg (17.5%) were the most frequently affected sites, reflecting the high mechanical load borne by these regions during sprinting, tackling, and rapid directional changes. The preponderance of cramps, strains, and contusions is similar to data from African tournaments [10,11], reinforcing the role of fatigue and contact as major mechanisms of injury. The relatively low proportion of ankle and knee injuries compared with other cohorts could be supported by under diagnosis or misclassification in the Cameroonian context where advanced imaging is still limited.

### Diagnosis and severity

Muscle and tendon injuries accounted for nearly 40% of all cases, aligning with global surveillance data where soft-tissue trauma predominates [7,8,20,22]. Notably, 20.5% of injuries were severe, a figure similar to those reported in elite competitions but with a different distribution of severity: muscle tears, fractures, concussions were severe, whereas minor soft-tissue conditions such as cramps or strains were less likely to cause time loss. The clustering of severe cases among goalkeepers (45.5%) emphasizes their exposure to high-impact and collision mechanisms, a pattern also observed in FIFA World Cup analyses [4]. Comparable severity across league tiers suggests that although incidence differs, the intrinsic nature of injuries sustained in both divisions is similar once they occur. This could be explained by the even physical nature and contact involved in African football.

### Timing and match demands

The late-match clustering of injuries during the final 30 minutes of play is one of the study’s most notable findings. This temporal pattern, also described in UEFA and AFCON surveillance [7,10], supports the hypothesis that cumulative fatigue, dehydration, and reduced neuromuscular control elevate injury risk as matches progress. More than half of all cramps occurred between 76–90 minutes, highlighting fatigue-related pathophysiology. Also given that we recorded only medical attention injuries, and that the playoffs tournament are very decisive some cramps may have been simulated. However, no formal protocol was used to distinguish physiological cramps from potential tactical behaviours. Match-day medical officers recorded all medical-attention events based on observable clinical signs and the player’s need for treatment, without attempting to adjudicate player intent. Consequently, while fatigue is a plausible explanation for the high late-match prevalence of cramps, any suggestion of tactical simulation should be interpreted cautiously as contextual speculation rather than a diagnostic conclusion. These results advocate for conditioning programs emphasizing endurance, recovery optimization, and in-game hydration strategies to mitigate late-match injury burden. The early occurrence of lacerations, mostly in defenders, further indicates the predominance of heavy contact mechanisms in the initial competitive phases of matches and the physical nature of football in Cameroon.

### Player position and functional demands

Midfielders and attackers accounted for nearly 70% of all injuries, reflecting their continuous involvement in both offensive and defensive play, high-intensity running, and frequent physical duels. Similar positional risk profiles have been reported in elite European cohorts [7,8] and African youth studies [11,12]. The positional clustering of injury types such as fatigue-related cramps in attackers and contact lacerations in defenders emphasizes the need for role-specific preventive interventions, including tailored workload management and position-specific strength conditioning.

### Implications and recommendations for injury prevention in african professional football

The markedly higher injury incidence in the lower-tier competition suggests that improved pitch quality, consistent pre-match warm-ups, and structured recovery protocols could substantially reduce injury rates. The predominance of fatigue-related and contact injuries supports the need for targeted prevention programs focusing on strength, endurance, and technique in tackling or aerial challenges. Also, the high prevalence of cramps during the ending minutes of the game should be questioned by policy makers to investigate simulation of cramps during this period as a time management technique. The comparable severity rates across divisions but greater incidence in Elite 2 highlights disparities in medical preparedness and access to physiotherapy key gaps that national federations and clubs should address. Implementing standardized medical staffing, precompetition screenings, and evidence-based prevention models such as FIFA’s “11+” program could have measurable benefits.

### Strengths and limitations

This study represents one of the few prospective injury surveillance projects in sub-Saharan African professional football. The use of standardized consensus based data collection forms and adherence to international consensus definitions enhances comparability with global datasets. However, several limitations must be acknowledged as only medical-attention injuries were recorded limiting total injury burden estimation. Injury severity should be interpreted as anticipated rather than confirmed time-loss, as post-tournament clinical follow-up of injured players was not feasible.

## Conclusion

This study demonstrates a high incidence of match-related injuries among professional footballers in Cameroon, particularly in lower-tier competitions. The predominance of lower-limb soft-tissue injuries, late-match clustering, and significant proportion of severe cases underline the urgent need for comprehensive injury prevention and medical readiness strategies. Context-specific adaptations of established prevention programs, investment in medical infrastructure, and continuous surveillance are crucial to enhancing player safety in African professional football.
